# Interval colorectal cancer rates after Hemoccult Sensa and survival by detection mode for individuals diagnosed with colorectal cancer in Winnipeg, Manitoba

**DOI:** 10.1371/journal.pone.0203321

**Published:** 2018-09-04

**Authors:** Kathleen M. Decker, Zoann Nugent, Pascal Lambert, Natalie Biswanger, Harminder Singh

**Affiliations:** 1 Department of Community Health Sciences, Rady Faculty of Medicine, University of Manitoba, Winnipeg, Manitoba, Canada; 2 Research Institute in Oncology and Hematology, CancerCare Manitoba, Winnipeg, Manitoba, Canada; 3 Epidemiology and Cancer Registry, CancerCare Manitoba, Winnipeg, Manitoba, Canada; 4 Department of Internal Medicine, University of Manitoba, Winnipeg, Manitoba, Canada; 5 Medical Oncology and Haematology, CancerCare Manitoba, Winnipeg, Manitoba, Canada; University of Kentucky, UNITED STATES

## Abstract

**Objective:**

To assess the performance of the Sensa fecal occult blood test (FOBT) in a population-based screening program.

**Setting:**

Manitoba, Canada.

**Methods:**

This historical cohort study included individuals 52 to 74 years of age diagnosed with colorectal cancer (CRC) from 2008 to 2013. CRCs were categorized by detection following a screening program FOBT (Sensa), non-program FOBT (non-Sensa), or no FOBT. Screening program CRCs were classified as program-detected, interval program, or non-compliant. Logistic regression was used to compare characteristics by detection mode. Cox regression adjusted for lead-time was used to examine the effect of detection mode on survival.

**Results:**

1,498 individuals were diagnosed with CRC; 132 (8.8%) had a screening program FOBT, 626 (41.8%) had a non-program FOBT, and 740 (49.4%) had no FOBT. Of the screening program FOBT CRCs, 72 were program-detected (54.5%), 42 were interval program (31.8%), and 18 were non-compliant (13.6%). Sensa interval cancer rate was 37.4% and sensitivity was 63.1% (95% Confidence Interval (CI): 54.3%-72.0%). The risk of death for individuals that had a non-program (Hazard ratio (HR) = 0.57, 95% CI:0.44–0.75) or a screening program FOBT (HR = 0.55, 95% CI:0.31–0.97) was lower than no FOBT. There was no significant difference in the risk of death for interval, non-compliant, and non-program CRCs compared to program-detected CRCs. Adjusting for lead time bias, sex, income quintile, tumour location, and age at diagnosis did not appreciably change the risk estimates.

**Conclusion:**

More than one-third of CRCs may not be detected by Sensa. There may be no difference in survival between CRC detected by Sensa and non-Sensa FOBTs.

## Introduction

Several observational studies have evaluated the impact of colorectal cancer (CRC) screening on population mortality and reported that that survival for screen-detected cancers is higher than symptomatically-detected cancers [1–7]. However, few adjusted for lead-time bias (screening leading to diagnosis earlier in the natural history of the disease without necessarily altering the natural history) in their analyses [2, 4]. Moreover, although guidelines continue to recommend the use of the newer guaiac-based fecal occult blood test (FOBT) Hemoccult II Sensa as one of the options for CRC screening, none of the trials that evaluated screening used the Sensa FOBT [8]. Thus, there are limited data on Sensa FOBT’s performance [9–11].

All Canadian provinces have implemented organized, population-based CRC programs [12]. Manitoba’s program (ColonCheck) began in August 2007. ColonCheck targets average risk individuals 50 to 74 years of age using the Sensa FOBT [13]. Guaiac-based FOBTs, including Sensa, detect the heme component of haemoglobin molecules because of the pseudoperoxidase activity of heme which converts guaiac to a blue colour when developer is added [14]. Unlike older guaiac FOBTs, Sensa has an enhancer added to the developer to permit detection of lower levels of peroxidase activity thereby increasing test sensitivity [14]. In Manitoba, ColonCheck mails an FOBT and instructions to eligible individuals every two years. Those with a positive result are sent for a follow-up colonoscopy. Manitoba residents may also complete a non-program FOBT that they receive when they visit their primary care provider (PCP). Non-program FOBTs are older, guaiac-based non-Sensa FOBTs. The objective of this research was to assess the performance of the Sensa FOBT in a population-based program. We aimed to determine the Sensa FOBT interval CRC rate and compare the characteristics and overall survival for individuals diagnosed with CRC using the screening program FOBT (Sensa) or a non-program FOBT (non-Sensa) to those with no FOBT. To determine if survival differs by detection mode, we also compared survival for three screening program FOBT sub groups: program-detected, interval program, and non-compliant CRCs.

## Materials and methods

### Study population

The province of Manitoba, located in central Canada, has a population of approximately 1.34 million (as of 2016) [15]. Two-thirds of the population lives in the capital city of Winnipeg. This study included all individuals 52 to 74 years of age (the age group targeted by the provincial screening program) diagnosed with CRC from 2008 to 2013 who lived in Winnipeg at diagnosis and for the preceding two years. Individuals who lived outside of Winnipeg were excluded because data for FOBTs in rural and northern areas of Manitoba are not completely captured in administrative health data. Individuals with a prior diagnosis of CRC and those that were resident in the province for less than two years were excluded.

### Description of data sources

Five data sources were used: the Manitoba Cancer Registry (MCR), the Manitoba Health Population Registry (MHPR), the Medical Claims Database (MCD), the ColonCheck registry, and Statistics Canada 2006 census data. The MCR is a population-based database that is legally mandated to collect, classify, and maintain accurate, comprehensive information about cancer cases including diagnosis date, histology, topography, stage, and treatment. The MCR was used to identify individuals diagnosed with CRC, area of residence, diagnosis age, tumour location, and death date.

The MHPR includes all provincial residents and is maintained by Manitoba Health, the publically-funded provincial health insurance agency. The MHPR contains demographic and migration information and a personal health identification number (PHIN) for all individuals which can be used to link provincial databases. The MHPR was used to determine provincial health coverage duration. The MCD, which includes claims filed by physicians and laboratories for payment of services, was used to identify individuals who had non-program FOBTs and colonoscopies after abnormal screening program FOBTs. The MHPR and the MCD have been validated for accuracy and have been used to study many health outcomes (19, 20).

Since FOBTs provided by the screening program are not included in MCD, the ColonCheck Registry was used to identify individuals who completed a screening program FOBT and the FOBT result. Statistics Canada 2006 census data were used to determine neighbourhood-level average household income (21). Average household income was categorized into quintiles from Q1 (the lowest income quintile) to Q5 (the highest income quintile) based on each individual’s area of residence. Previous reports have found that census-derived area-based income measures are reasonable proxies for individual-level income (22–24). To protect confidentiality, linkages were performed via scrambled PHINs using anonymous versions of all databases and no patients were contacted. The study was approved by the University of Manitoba’s Health Research Ethics Board and Manitoba Health’s Information Privacy Committee.

### Definitions of outcomes and study measures

CRCs were classified by detection mode as screening program FOBT, non-program FOBT, or no FOBT. Screening program FOBT detected CRCs included individuals that completed a screening program FOBT (Sensa) performed within two years preceding the CRC diagnosis. Non-program (non-Sensa) FOBTs included individuals that had a non-program (non-Sensa) FOBT performed in the two years preceding a CRC diagnosis and no screening program FOBT in the same time period (i.e., the non-Sensa FOBT was completed through their PCP). Individuals diagnosed with CRC that had no FOBT in the two years prior to the CRC diagnosis date were classified as no FOBT.

We further classified individuals that had a screening program FOBT as either program-detected, interval program, or non-compliant. Program detected CRCs included individuals that had an abnormal screening program FOBT, subsequent colonoscopy within a year, and then a CRC diagnosis within 90 days of the colonoscopy. Interval program CRCs included individuals that had 1) a normal screening program FOBT within the 2 years preceding a CRC diagnosis or 2) an abnormal screening program FOBT followed by a subsequent colonoscopy within a year of FOBT, and then a CRC diagnosed greater than 90 days after the colonoscopy (i.e., missed at colonoscopy). Non-compliant CRCs included individuals that had 1) a normal screening program FOBT more than 2 years preceding a CRC diagnosis and no other FOBT within those 2 years, 2) an abnormal screening program FOBT with no colonoscopy within the subsequent year and CRC diagnosed within the subsequent 2 years, or 3) an indeterminate screening program FOBT with no repeat FOBT and a CRC diagnosed within 2 years of the indeterminate FOBT.

Right-sided CRCs were defined as those occurring in the cecum, ascending colon, hepatic flexure, and transverse colon. Left-sided CRCs were defined as those occurring in the splenic flexure, descending colon, and sigmoid colon.

### Statistical analysis

Fisher’s exact tests using Monte Carlo estimation, Kruskal-Wallis test, and logistic regression were used to compare the characteristics of individuals diagnosed with CRC by detection mode. Cox regression was used to examine the impact of detection mode on the risk of death using bootstrapping with 1,000 resamples to obtain more reliable estimates. We examined models adjusted for income quintile, sex, tumour location, and age at diagnosis. Income quintile and sex were included to attempt to control for selection bias [16, 17]. Age at diagnosis and tumour site were included in the final model since both may be related to screening and cancer (i.e., individuals that are screened are more likely to be diagnosed at a younger age or with a left-side CRC) [1, 2, 5]. Stage was not included in the models because stage is a mediating factor between screening and survival which would result in underestimating the effect of screening.

Follow-up time was defined as the time from the CRC diagnosis date to death date, migration date, or the end of the follow-up period (November 30, 2015). To factor in lead-time bias, we applied the correction developed by Duffy *et al*. to all screening program detected and to 80% of non-program detected CRCs [18]. Although the FOBT is not recommended as a diagnostic test, it is estimated that approximately 20% of non-program FOBTs are performed for purposes other than screening asymptomatic individuals [19, 20]. Therefore, 80% of the non-program group was randomly selected and the lead-time bias correction was applied to this group. This process was repeated 1,000 times and the results from those models were summarized using Rubin’s rules [21].

Since the mean sojourn time (when the tumour is asymptomatic but screen-detectable) has been estimated to range from three to six years, we calculated survival for 0, 2 and 5 year sojourn times [22–26]. Schoenfeld residual plots were used to test the proportional hazards assumption for each variable. Linearity of age at diagnosis was verified using natural cubic splines. Statistical tests were carried out using SAS version 9.4 (SAS Institute Inc., Cary, North Carolina). All tests were 2-sided and p values <0.05 were considered statistically significant.

## Results

**Fig 1:**
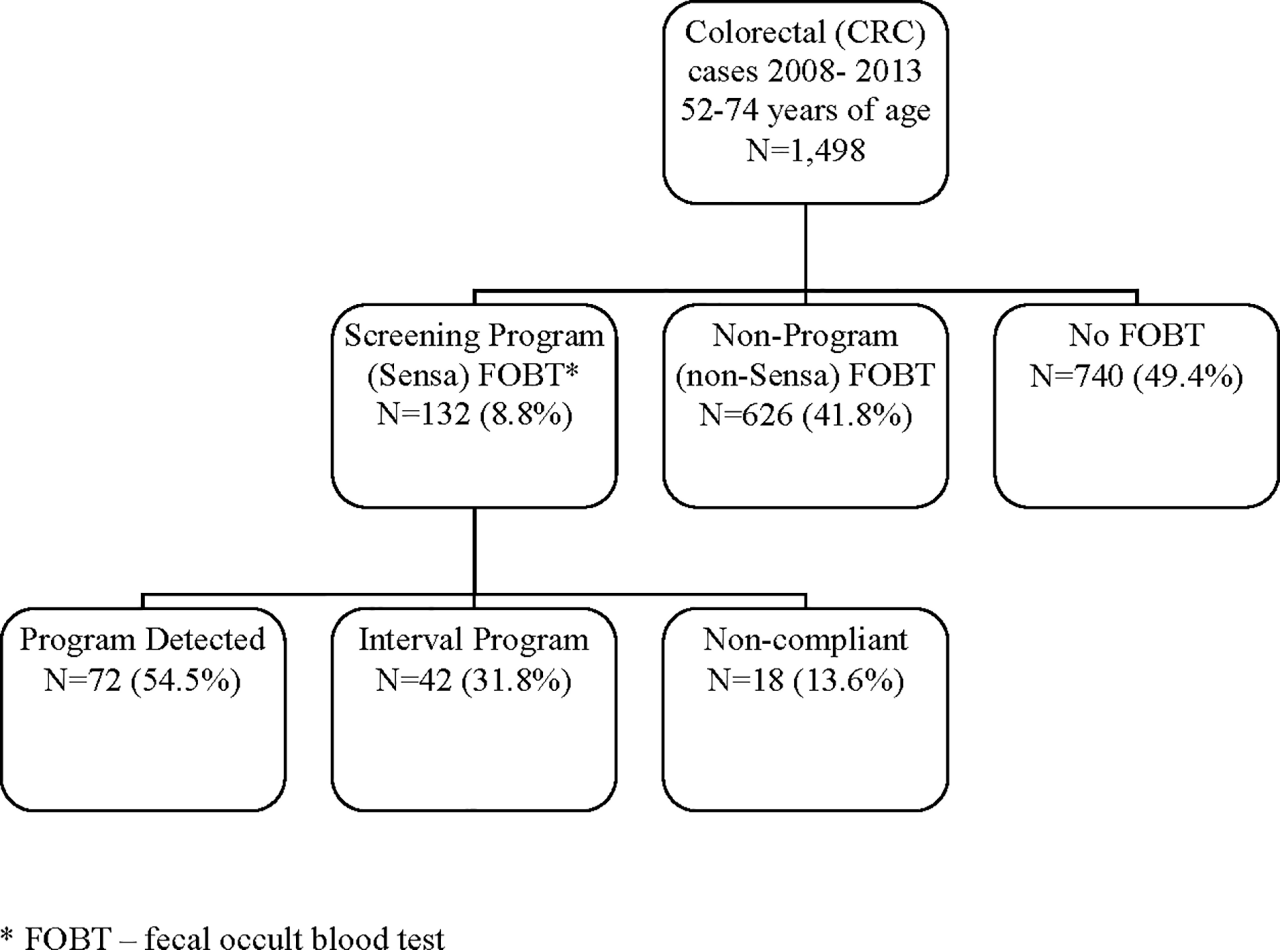
Individuals 52 to 74 years of age diagnosed with colorectal cancer (CRC) by detection mode, 2008–2013, Winnipeg, Manitoba.

From 2008 to 2013, 1,498 individuals 52 to 74 years of age who lived in Winnipeg were diagnosed with CRC. Overall, 626 people (41.8%) diagnosed with CRC had a non-program FOBT, 740 (49.4%) had no FOBT, and 132 (8.8%) had a screening program FOBT. Of the individuals that completed a screening program FOBT, 72 were classified as program-detected (54.5%), 42 were interval program (31.8%), and 18 were non-compliant (13.6%) (Fig 1).

The interval CRC proportion was 0% in 2008, 10.0% in 2009, 16.7% in 2010, 12.9% in 2011, 52.8% in 2012, and 41.5% in 2013. Excluding non-compliant CRCs, the Sensa FOBT interval CRC rate was 36.8% (95% Confidence Interval (CI) 29.0%, 45.7%) and sensitivity for CRC detection was 63.1% (95% CI 54.3%, 72.0%).

The characteristics of the individuals diagnosed with CRC by detection mode are shown in Table 1.

**Table 1:** Number and percentage of individuals diagnosed with colorectal cancer by detection mode, sex, age group, stage at diagnosis, income quintile, diagnosis year, and tumour location, 2008–2013, Winnipeg, Manitoba.

		Detection Mode					
				Screening program FOBT			
		Non-program FOBT	No FOBT	Program	Interval	Non-compliant	P value
		N (%)*	N (%)	N (%)	N (%)	N (%)	
** **	**N**	626	740	72	42	18	
**Sex**	**Male**	373 (59.6)	451 (60.9)	46 (63.9)	26 (61.9)	6 (33.3)	0.203
	**Female**	253 (40.4)	289 (39.1)	26 (36.1)	16 (38.1)	12 (66.7)	
**Age**	**Mean**	65.0	65.0	64.0	64.9	65.6	0.85
**Stage**	**I**	146 (24)	124 (17.3)	31 (43.1)	10 (23.8)	6 (33.3)	**< .0001**
	**II**	170 (27.9)	163 (22.8)	16 (22.2)	13 (31.0)	s	
	**III**	196 (32.2)	239 (33.4)	20 (27.8)	11 (26.2)	s	
	**IV**	97 (15.9)	190 (26.5)	s	8 (19.1)	s	
	**Missing**	17 (2.7)	24 (3.2)	0	0	0	
**Income quintile**	**Q1 (lowest)**	102 (16.3)	171 (23.1)	15 (20.8)	s	s	**0.001**
	**Q2**	118 (18.9)	173 (23.4)	13 (18.1)	6 (14.3)	6 (33.3)	
	**Q3**	132 (21.1)	139 (18.8)	15 (20.8)	8 (19.1)	s	
	**Q4**	141 (22.5)	132 (17.8)	13 (18.1)	8 (19.1)	7 (38.9)	
	**Q5 (highest)**	133 (21.3)	125 (16.9)	16 (22.2)	16 (38.1)	s	
**Diagnosis year**	**2008**	113 (18.1)	142 (19.2)	s	0 (0)	0 (0)	**<0.0001**
	**2009**	100 (16.0)	133 (18.0)	9 (12.5)	s	0 (0)	
	**2010**	106 (16.9)	121 (16.4)	10 (13.9)	s	s	
	**2011**	114 (18.2)	133 (18.0)	24 (33.3)	s	s	
	**2012**	97 (15.5)	100 (13.5)	13 (18.1)	19 (45.2)	s	
	**2013**	96 (15.3)	111 (15.0)	13 (18.1)	16 (38.1)	11 (61.1)	
**Tumour Location**	**Right-sided (Proximal)**	196 (31.3)	251 (33.9)	17 (23.6)	14 (33.3)	8 (44.4)	**0.014**
	**Left-sided (Distal)**	182 (29.1)	186 (25.1)	35 (48.6)	10 (23.8)	s	
	**Rectum**	248 (39.6)	303 (41.0)	20 (27.8)	18 (42.9)	7 (38.9)	

Notes

* Column percentages

Q1 = income quintile level 1 (lowest) ($14,640 to $42,407), Q2 = income quintile level 2 ($42,463 to $54,663), Q3 = income quintile level 3 ($54,696 to $68,132), Q4 = income quintile 4 ($68,140 to $87,201), Q5 = income quintile 5 (highest) ($87,214 to $406,531). Tumour location–right-sided (proximal) includes the cecum, ascending colon, hepatic flexure, and transverse colon, left-sided (distal) includes splenic flexure, descending colon, and sigmoid colon, rectum includes rectosigmoid junction. FOBT–fecal occult blood test. Only one FOBT was counted every two years. s = suppressed to protect patient anonymity when the numbers were <6.

A higher percentage of individuals diagnosed with a program-detected CRC were diagnosed at stage I (43.1%) and were more likely to be diagnosed in the left colon (48.6%). The proportion of program-detected CRCs and those that occurred following a non-program FOBT remained fairly stable over time. A higher proportion of CRCs that had no FOBT occurred in the lower two income groups.

After adjusting for sex, income quintile, and age, right-sided CRCs were more likely to be non-program FOBT detected (Odds Ratio (OR) = 2.09, 95% CI 1.11, 3.90), no FOBT (OR = 2.70, 95% CI 1.46, 4.99), or interval (OR = 3.42, 95% CI 1.18, 9.89) compared to program-detected CRCs (Table 2). Rectal tumours were also more likely to be non-program FOBT detected (OR = 2.39, 95% CI 1.33, 4.29), no FOBT (OR = 2.84, 95% CI 1.59, 5.09), or interval (OR = 4.48, 95% CI 1.57, 12.80) compared to program-detected CRCs.

**Table 2:** Logistic regression analysis of the characteristics associated with a colorectal cancer diagnosis following a non-program fecal occult blood test (FOBT), no FOBT, or an interval colorectal cancer compared to a program-detected colorectal cancer.

Characteristics		Univariable				Multivariable		
		OR		95% CI	p value	OR	95% CI	p value
**Non-program FOBT**								
**Sex**	**Male**	0.83	0.50–1.88		0.4800	0.82	0.49–1.37	0.4500
	**Female**	1.00	-			1.00	-	
**Location**	**Right-sided (Proximal)**	2.22	1.20–4.10		**0.0139**	2.09	1.11–3.90	**0.0057**
	**Rectum**	2.39	1.33–4.27			2.39	1.33–4.29	
	**Left-side** **(Distal)**	1.00	-			1.00	-	
**Income quintile**	**Q1**	0.82	0.39–1.73		0.8400	0.76	0.36–1.64	0.8000
	**Q2**	1.09	0.50–2.37			1.04	0.47–2.24	
	**Q3**	1.06	0.50–2.23			1.06	0.50–2.24	
	**Q4**	1.31	0.61–2.82			1.27	0.58–2.77	
	**Q5**	1.00	-			1.00	-	
**Age**		1.02	0.99–1.06		0.2400	1.02	0.98–1.06	0.3000
**No FOBT**								
**Sex**	**Male**	0.88	0.53–1.46		0.6200	0.90	0.54–1.51	0.6900
	**Female**	1.00	-			1.00	-	
**Location**	**Right-sided (Proximal)**	2.78	1.51–5.11		**0.0002**	2.70	1.46–4.99	**0.0003**
	**Rectum**	2.85	1.60–5.09			2.84	1.59–5.09	
	**Left-sided (Distal)**	1.00	-			1.00	-	
**Income quintile**	**Q1**	1.46	0.70–3.06		0.7100	1.33	0.63–2.84	0.8300
	**Q2**	1.70	0.79–3.67			1.59	0.73–3.47	
	**Q3**	1.19	0.56–2.50			1.18	0.55–2.51	
	**Q4**	1.30	0.60–2.81			1.22	0.56–2.68	
	**Q5**	1.00	-			1.00	-	
**Age**		1.02	0.99–1.05		0.2800	1.02	0.99–1.05	0.2600
**Interval**								
**Sex**	**Male**	0.92	0.42–2.02		0.8300	0.79	0.33–1.89	0.6000
	**Female**	1.00	-			1.00	-	
**Location**	**Right-sided (Proximal)**	2.88	1.06–7.81		**0.0370**	3.42	1.18–9.89	**0.0120**
	**Rectum**	3.15	1.22–8.13			4.48	1.57–12.80	
	**Left-sided (Distal)**	1.00	-			1.00	-	
**Income quintile**	**Q1**	0.27	0.07–0.98		0.3400	0.19	0.05–0.68	0.1400
	**Q2**	0.46	0.14–1.52			0.36	0.10–1.33	
	**Q3**	0.53	0.18–1.61			0.48	0.15–1.55	
	**Q4**	0.62	0.20–1.89			0.42	0.13–1.39	
	**Q5**	1.00	-			1.00	-	
**Age**		1.02	0.96–1.08		0.5100	1.02	0.96–1.09	0.5100

Notes

Q1 = income quintile level 1 (lowest), Q2 = income quintile level 2, Q3 = income quintile level 3, Q4 = income quintile 4, Q5 = income quintile 5 (highest). Tumour location–Right-sided (proximal) includes the cecum, ascending colon, hepatic flexure, and transverse colon, left-sided (distal) includes splenic flexure, descending colon, and sigmoid colon, rectum includes rectosigmoid junction. FOBT–fecal occult blood test. OR–Odds Ratio. CI–confidence interval

There were 167 deaths in the non-program FOBT group, 367 in the no FOBT group, 13 in the program-detected group, 9 in the interval program group, and fewer than six deaths in the non-compliant group. The median follow-up time was 3.5 years. Table 3 shows the risk of death adjusted for gender, income quintile, age, and tumour location for individuals diagnosed with CRC that had a non-program or a screening program FOBT compared to those who had no FOBT (the reference group).

**Table 3:** Risk of death for individuals diagnosed with colorectal cancer (CRC) after a non-program provided fecal occult blood test (FOBT) or a screening program FOBT compared to individuals diagnosed with CRC after no FOBT (reference group) adjusted for gender, income quintile, age, and tumour location.

Detection Mode	No sojourn time			2 year sojourn time			5 year sojourn time		
	HR	95% CI	p value	HR	95% CI	p value	HR	95% CI	p value
**Non-program FOBT**	0.44	0.37–0.53	**<0.0001**	0.57	0.44–0.75	**<0.0001**	0.71	0.54–0.93	0.8388
**Screening program FOBT**	0.37	0.25–0.55	**<0.0001**	0.52	0.31–0.97	**0.0374**	0.69	0.45–1.38	0.3966
**No FOBT**	1.00	-	-	1.00	-	-	1.00	-	-

Notes

Sojourn time is the interval during which the cancer is potentially detectable through screening but not yet clinically diagnosed. FOBT–fecal occult blood test. HR–Hazard ratio. CI–Confidence Interval

After adjusting for lead-time bias (sojourn time of 2 years), the risk of death for individuals that had a non-program or a screening program FOBT was half of those that had no FOBT (non-program FOBT HR = 0.57, 95% CI 0.44, 0.75, screening program FOBT HR = 0.55, 95% CI 0.31, 0.97). At a sojourn time of five years, the HR of death for individuals who had completed a non-program FOBT was 0.71 (95% CI 0.54, 0.93) and the HR for those that had a screening program FOBT was 0.78 (95% CI 0.45, 1.38).

Table 4 provides the risk of death by sojourn time comparing interval program and non-compliant CRCs to program-detected CRCs (the reference group) for 3 models (unadjusted, adjusted for sex and income quintile, and adjusted for sex, income quintile, and tumour location, and age at diagnosis) using bootstrapping to increase reliability.

**Table 4:** Risk of death by sojourn time unadjusted (model 1), adjusted for gender and income quintile (model 2), and adjusted for gender, income quintile, age, and tumour location (model 3).

	No sojourn time						2 year sojourn time			5 year sojourn time		
Detection Mode	HR	95% CI			p value		HR	95% CI	p value	HR	95% CI	p value
**Model 1 (unadjusted)**												
**Interval**	1.49	0.43–5.23			0.5305		1.66	0.47–5.88	0.4287	1.77	0.51–6.11	0.3660
**Non-compliant**	1.53	0.28–8.29			0.6231		1.62	0.29–9.10	0.5808	1.71	0.32–9.20	0.5309
**Non-program FOBT**	1.67	0.75–3.72			0.2051		1.45	0.63–3.33	0.3843	1.28	0.56–2.92	0.5543
**No FOBT**	3.50	1.60–7.67			**0.0017**		2.59	1.14–5.91	**0.0231**	1.85	0.82–4.19	0.1395
**Program-detected**	1.00	-			-		1.00	-	-	1.00	-	-
**Model 2 (adjusted for sex and income quintile)**												
**Interval**	1.65		0.46–5.93			0.4416	1.80	0.50–6.53	0.3687	1.91	0.54–6.74	0.3157
**Non-compliant**	1.56		0.27–8.85			0.6163	1.77	0.31–10.05	0.5204	1.87	0.34–10.25	0.4689
**Non-program FOBT**	1.71		0.73–3.98			0.2150	1.45	0.64–3.27	0.3757	1.28	0.57–2.87	0.5489
**No FOBT**	3.42		1.48–7.89			**0.0040**	2.51	1.13–5.61	**0.0245**	1.79	0.81–3.97	0.1521
**Program-detected**	1.00		-			-	-	-	-	-	-	-
**Model 3 (adjusted for sex, income quintile, tumour location, and age at diagnosis)**												
**Interval**	1.53			0.42–5.62		0.5180	1.71	0.47–6.24	0.4129	1.82	0.51–6.46	0.3524
**Non-compliant**	1.44			0.26–8.08		0.6814	1.59	0.28–9.05	0.6019	1.68	0.31–9.14	0.5503
**Non-program**	1.49			0.64–3.47		0.3598	1.36	0.57–3.22	0.4875	1.20	0.52–2.81	0.6682
**No FOBT**	3.22			1.40–7.42		**0.0061**	2.35	1.00–5.49	**0.0488**	1.68	0.73–3.89	0.2247
**Program-detected**	1.00			-		-	1.00	-	-	1.00	-	-

Notes

FOBT–fecal occult blood test. HR–Hazard Ratio. CI–confidence interval

After adjusting for lead-time bias using a sojourn time of two years, the risk of death was non-significantly higher for individuals with an interval program (HR = 1.66, 95% CI 0.47, 5.88), non-compliant (HR = 1.62, 95% CI 0.29, 9.10), or non-program FOBT (HR = 1.45, 95% CI 0.63, 3.33). The risk of death for individuals who did not have a FOBT was twice as high as program-detected CRCs (HR = 2.59, 95% CI 1.14, 5.91). Adjustment for sex, income quintile, tumour location, and age at diagnosis did not appreciably change the risk estimates. At a sojourn time of five years, the risk of death was not significantly higher for individuals diagnosed through the other detection modes compared to program-detected CRCs.

## Discussion

This study shows the beneficial impact on survival for individuals who had a screening program or non-program FOBT compared to individuals who had no FOBT after adjustment for lead time bias. However, the Sensa interval CRC rate in this study was 36.8% with a sensitivity of 63.1%. The guaiac Hema-screen FOBT (an older guaiac) pilot in Scotland found an interval CRC rate (diagnosed within two years of a negative FOBT out of all individuals screened) of 31.2% in the first round of screening, 47.7% in the second round, and 58.9% in the third round [27]. A summary by York University in 2007 found that the sensitivity of Sensa for the detection of CRC ranged from 62% to 79% [28]. A 2016 review for the US Preventive Services Task Force (USPSTF) (n = 15,969) found that Sensa sensitivity ranged from 61.5% (95% CI 35.0, 83.5) to 79.4% (95% CI 63.8, 90.3) [29]. Our results suggest that the current sensitivity of Sensa in real-world programmatic CRC screening may be lower than that reported in some of the prior studies and not much higher than lower-sensitivity, older guaiac FOBTs [30, 31]. In comparison, a meta-analysis by Lee *et al*. found that the sensitivity from 19 studies of fecal immunochemical tests (FIT) was 79% (95% CI, 69%, 86%) [32].

Prior observational studies that have examined the impact of FOBT screening on survival have also found improved survival for screen-detected CRCs. Brenner *et al*. found a 46% reduction in overall survival among FOBT program-detected CRCs and a 50% reduction among colonoscopy-detected CRC patients 50–79 years of age compared to those diagnosed symptomatically in Germany from 2003 to 2010 [1]. Higher overall survival was also found in England when comparing individuals 60 to 69 years of age diagnosed with a screen-detected CRC (using Hema-screen, an earlier FOBT version) to those diagnosed symptomatically from 2002 to 2010 [5, 6]. In Australia, FOBT screen-detected CRCs among individuals 50 to 70 years of age from 2006 to 2013 had a significantly lower overall death rate compared to symptomatic patients [7]. However, these studies did not adjust for lead-time bias.

In the present study, the risk of death for individuals that had no FOBT prior to diagnosis compared to program-detected CRC was three times higher when unadjusted for lead-time bias. The risk decreased when adjusted for lead-time bias using a sojourn time of two years and five years. Therefore, the results of observational studies that did not adjust for lead-time bias likely overestimated the benefits of FOBT screening in real-world practice.

Two prior observational analyses of screening survival did adjust for lead-time bias. Lindegjerg *et al*. examined overall survival for individuals diagnosed with CRC in Denmark from 2005 to 2008 [4]. The risk of death for the FOBT screening group was 70% lower than the unscreened group. An Australian study compared the survival of individuals 50 to 59 years of age invited to be screened using a FIT to those not invited from 2006 to 2008 [2]. CRC death was 13% lower in the invited group compared to the never-invited group. When program-detected CRCs were compared to CRCs among non-responders, the risk of death was over twice as great.

Our study also found no significant difference in risk of death for interval program CRCs compared to program-detected CRCs after controlling for lead-time bias. The Australian National Bowel Cancer Program also found that the survival for individuals with an interval program CRC was not significantly different than program-detected CRCs after adjusting for lead-time bias [2]. However, Lindebjerg *et al*. found a significantly higher risk of death for CRCs diagnosed after a negative FOBT (interval program CRCs) compared to program-detected CRCs adjusted for lead-time bias [4]. The lack of significance in our study may be due to the small number of deaths in the interval program CRC group and a lack of power to detect a significant difference.

We found no significant difference in risk of death for individuals diagnosed after a non-program FOBT compared to those diagnosed with a program-detected CRC. However, program-detected CRCs were more likely to be left-sided than those diagnosed after a non-program FOBT. Additionally, CRCs diagnosed after a non-program FOBT had similar characteristics to CRCs diagnosed after no FOBT (stage at diagnosis, tumour location, and age at diagnosis). Some of the difference between program-detected CRCs and those found after a non-program FOBT could be related to use of non-program FOBTs for symptom evaluation. We assumed that 20% of non-programs were completed by symptomatic individuals [19, 20]. In case the rate was higher, we ran a sensitivity analysis that increased the estimate to 50% symptomatic; results of survival analyses were similar (data not shown).

Unlike previous studies, we did not include stage at diagnosis in the survival analyses [1, 3]. Since stage is a mediating factor in the causal pathway between screening and survival, including stage means that any observed survival advantage due to screening cannot be isolated. We believe studies that included stage in the analysis likely underestimate the survival advantage due to screening. Finally, the higher proportion of individuals diagnosed with CRC after no FOBT in the lower two income groups highlights the on-going income disparities in CRC outcomes and the need for programs to focus screening among disadvantaged groups.

This study has strengths and weaknesses. Although we used data from previously validated population-based administrative health databases, this was an observational study and the potential for residual confounding by unmeasured or unrecognized factors exists [33–36]. The program FOBT participants are recruited through mailed packages from the program, whereas non-program FOBT participants are recruited through physician offices; whether this results in a participation bias is not known. While not recommended in Manitoba for average risk individuals, some of the individuals classified as having no FOBT before diagnosis may have been diagnosed through screening colonoscopy because they were at a higher risk for CRC or due to patient/physician preference. This may have reduced the magnitude of the observed benefit. In addition, we were not able identify interval program CRCs in the non-program group since we did not have results of the non-program FOBTs. Therefore, we could not calculate sensitivity for non-program FOBTs. We found a higher risk of death for interval program and non-compliant CRCs compared to program-detected CRC but the small number of deaths in both interval program and non-compliant CRC groups limited the precision of the analysis and data interpretation. Additional studies with a larger samples size are required. Finally, although we attempted to adjust for lead time bias, we did not adjust for length time bias which may lead to an exaggerated survival benefit attributed to screening.

## Conclusions

In conclusion, this study found a significant survival advantage for individuals diagnosed with CRC following a screening or a non-program FOBT compared to those who had no FOBT. The results of this study reinforce the importance of screening for CRC regardless of whether an FOBT is performed through an organized program or through an individual’s PCP. However, the sensitivity of Sensa in a real-world setting is at the lower end of the range previously reported and similar to that reported earlier for older guaiac FOBTs. This finding should prompt existing screening programs that use Sensa or older guaiac FOBTs to consider using alternate tests such as FIT and provide further justification for emerging screening programs to choose a FIT.
